# Targeting Cytoprotective Autophagy to Enhance Anticancer Therapies

**DOI:** 10.3389/fonc.2021.626309

**Published:** 2021-02-25

**Authors:** Malina Xiao, Alice Benoit, Meriem Hasmim, Caroline Duhem, Guillaume Vogin, Guy Berchem, Muhammad Zaeem Noman, Bassam Janji

**Affiliations:** ^1^ Tumor Immunotherapy and Microenvironment (TIME) Group, Department of Oncology, Luxembourg Institute of Health (LIH), Luxembourg City, Luxembourg; ^2^ Department of Hemato-oncology, Centre Hospitalier du Luxembourg, Luxembourg City, Luxembourg; ^3^ Université de Lorraine - UMR 7365, Ingénierie Moléculaire et Physiopathologie Articulaire (IMoPA), Vandoeuvre-lès-Nancy, France; ^4^ Centre François Baclesse, Esch-sur-Alzette, Luxembourg

**Keywords:** autophagy, cancer resistance, chemotherapy, radiotherapy, targeted therapy, immunotherapy

## Abstract

Autophagy is a highly regulated multi-step process that occurs at the basal level in almost all cells. Although the deregulation of the autophagy process has been described in several pathologies, the role of autophagy in cancer as a cytoprotective mechanism is currently well established and supported by experimental and clinical evidence. Our understanding of the molecular mechanism of the autophagy process has largely contributed to defining how we can harness this process to improve the benefit of cancer therapies. While the role of autophagy in tumor resistance to chemotherapy is extensively documented, emerging data point toward autophagy as a mechanism of cancer resistance to radiotherapy, targeted therapy, and immunotherapy. Therefore, manipulating autophagy has emerged as a promising strategy to overcome tumor resistance to various anti-cancer therapies, and autophagy modulators are currently evaluated in combination therapies in several clinical trials. In this review, we will summarize our current knowledge of the impact of genetically and pharmacologically modulating autophagy genes and proteins, involved in the different steps of the autophagy process, on the therapeutic benefit of various cancer therapies. We will also briefly discuss the challenges and limitations to developing potent and selective autophagy inhibitors that could be used in ongoing clinical trials.

## Introduction

**Figure 1:**
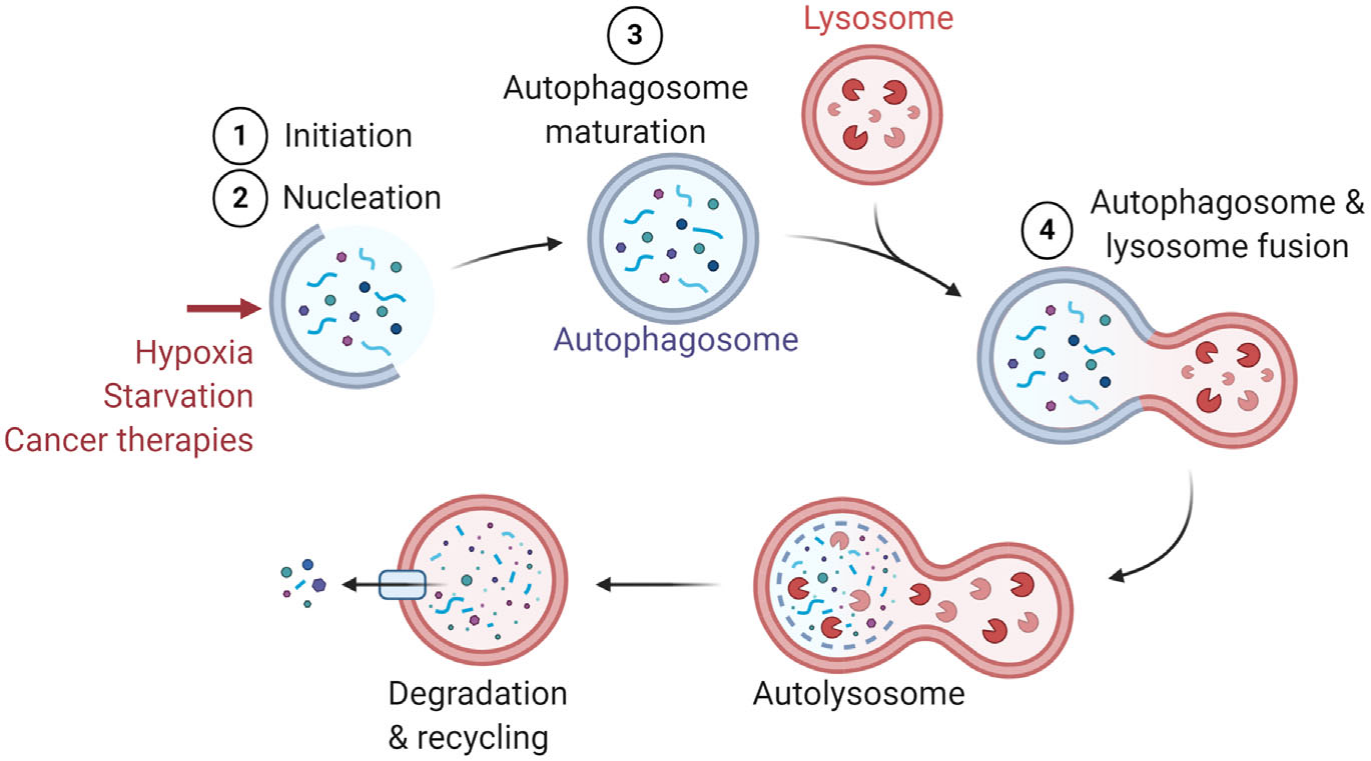
General presentation of the major steps of autophagy. Several stimuli have been identified to induce autophagy such as hypoxia, starvation, and cancer therapies. The major steps of autophagy are: 1) Initiation, 2) Nucleation, 3) Maturation, and 4) Fusion with lysosome for the degradation and recycling of autophagosome constituents.

Macroautophagy (referred to as autophagy) was first described in 1966 as a cellular process that occurs at the basal level in all cells (1). Autophagy relies on the formation of double-membraned vesicles known as autophagosomes, leading to the degradation of their cargo, such as damaged proteins or organelles (2). The autophagy process involves more than thirteen autophagy-related (ATG) proteins and requires the following major steps: (i) Initiation, (ii) Nucleation, (iii) Maturation, and (iv) Fusion with lysosome for cargo degradation (**Figure 1**). Originally described as a bulk degradation process, autophagy is now described as a highly selective degradation mechanism for the recycling of cellular components. Autophagy can be activated as an adaptive cellular response to external stimuli such as hypoxia, starvation, and different cancer therapies and therefore considered as a cytoprotective mechanism (1, 3–5).

Autophagy is activated under nutrient deprivation or starvation condition, which resulted in a decrease of mTOR activity and an increase of Unc-51 like autophagy activating kinase 1 (ULK1) activation. Activated ULK1 is subsequently dissociated from the 5’ adenosine monophosphate-activated protein kinase (AMPK), resulting in autophagy activation (6, 7). In addition to starvation, autophagy can also be activated in the tumor microenvironment by hypoxia through the hypoxia-inducible factor 1-α (HIF-α). The accumulation of HIF-1α in hypoxic cells activates the expression of BNIP/BNIP3L, which subsequently dissociates the complex between Bcl-2 and Beclin-1 (BECN1) to activate autophagy (8).

Autophagy was primarily considered as a tumor suppressive mechanism. Such a role was supported by studies showing that targeting BECN1, ATG5, and ATG7 promotes tumor initiation (9–11). In particular, evidences have demonstrated that Everolimus, an mTOR inhibitor and analogue of rapamycin, significantly increases mice survival in acute lymphoblastic leukemia in combination with Vincristine (12, 13). Conversely, many groups highlight the tumor supportive role of autophagy by showing its role in promoting tumor cell survival and growth in multiple tumor types (14, 15). The consensus appears to be that autophagy plays double-edged sword in suppressing tumor initiation and in promoting the survival of established tumors (16). Indeed, experimental evidence points at autophagy as a mechanism involved in cancer cell resistance to various therapies, such as chemotherapy, radiotherapy, targeted therapy, photodynamic therapy-induced apoptosis, and immunotherapy (17–21). Despite the complex interplay between the tumor suppressive and supportive role of autophagy in cancer (14), the vast majority of the clinical trials have focus on inhibiting autophagy with chloroquine (CQ) and hydroxychloroquine (HCQ) either alone or in combination with anticancer therapies (22). Therefore, autophagy inhibition has been suggested as a strategy to improve cancer therapies and has been considered in multiple clinical trials. Autophagy inhibitors have been classified according to their action on the major steps of autophagy and numerous preclinical studies have evaluated the therapeutic benefit of inhibiting autophagy.

**Figure 2:**
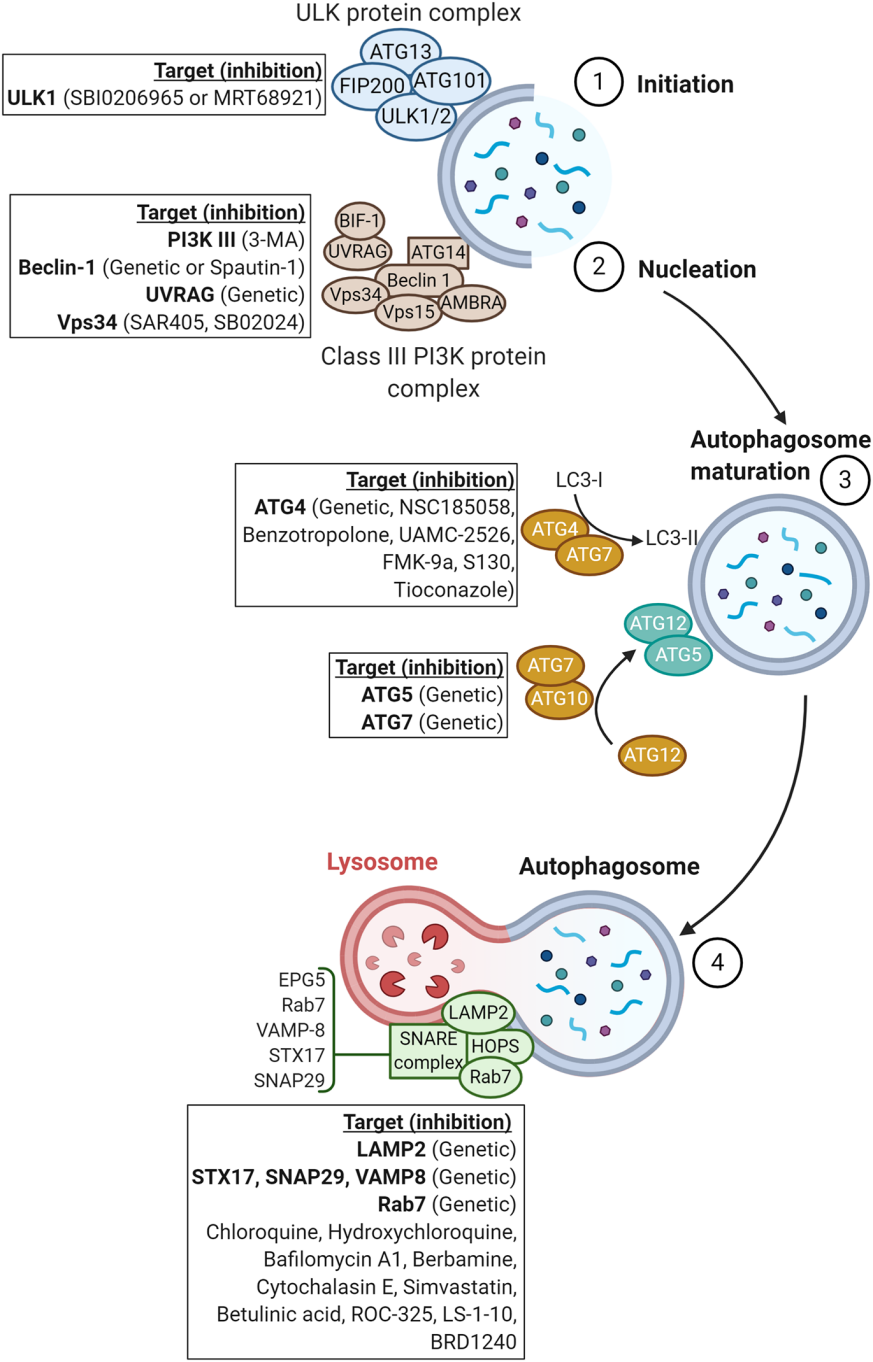
Schematic representation of proteins involved in the major steps of autophagy. Genetic or pharmacological approaches targeting proteins involved in each step of autophagy are reported in squares.

In this review, we will summarize the impact of inhibiting the different steps of autophagy, either pharmacologically or genetically by silencing or knocking down autophagy-associated genes (**Figure 2**), and describe how autophagy can be leveraged to improve the therapeutic benefit of current cancer therapies and elicit a synergistic effect with antineoplastic agents.

## Impact of Inhibiting the Initiation and Nucleation Steps on Cancer Therapies

The first step of the autophagy process, so-called the initiation step, involves the ULK protein complex including ATG13, ATG101/ULK1/2, and FIP200 (2, 23). The initiation step of autophagy facilitates the recruitment of the class III PI3K or VPS34 complex containing BECN1, VPS34, regulatory subunit 4 (VPS15/P150), activating molecule in BECN1-regulated autophagy protein 1 (AMBRA), UV radiation resistance-associated gene protein (UVRAG), BIF1, and ATG14L (2, 23) to the newly formed “phagophore”. The recruitment of the class III PI3K constitutes the nucleation step. In this section, we will summarize the different drugs and/or strategies used to target the initiation and nucleation steps and briefly discuss data available on their efficacy in pre-clinical tumor mouse models.

### Inhibition of ULK1

Several long noncoding RNAs (lncRNAs) have been reported to induce tumor chemoresistance to 5-fluorouracil (5-FU) such as lncRNA H19 and lncRNA small nucleolar RNA host gene 6 (SNHG6) in colorectal cancer (24, 25). SNHG6 promotes resistance of mice bearing RKO colon tumors to 5-FU. Furthermore, investigations showed that SNHG6 induced ULK1-dependent autophagy *via* sponging miR-26a-5p (25).

SBI0206965 is a highly selective inhibitor of ULK1 kinase (26) and it has been reported to sensitize NSCLC cells and acute myeloid leukemia (AML) cells to cisplatin- and daunorubicin-based chemotherapy, respectively, by decreasing cancer cell viability (27, 28). In pancreatic ductal adenocarcinoma (PDAC) cells, combining extracellular signal-regulated kinase (ERK) inhibitors with inhibitors of ULK1 complex (SBI0206965 or MRT68921) or with spautin-1, a specific inhibitor of two ubiquitin-specific peptidases USP10 and USP13 that control BECN1 degradation (29), decreased cell proliferation relative to ERK inhibitors alone (30). Recently, Chen et al. demonstrated that simultaneous inhibition of ULK1 (MRT68921) with NUAK1 (also known as ARK1) induces apoptosis in various cancer types (31).

### Inhibition of Class III PI3K

Pre-treatment with class III PI3K inhibitors, such as 3-Methyladenine (3-MA), showed a significant improvement of the sensitivity of MDA-MB-231 and HBL-100 breast cancer cells to ionizing radiation (IR) despite an apparent low level of basal autophagy in HBL-100 cells (32). The therapeutic benefit of combining IR and 3-MA was observed in a xenograft esophageal squamous cell carcinoma mice model *in vivo* with a significant decrease in tumor volume relative to single treatment (33). Moreover, it has been demonstrated that 3-MA in combination with docetaxel, a semi-synthetic analog of paclitaxel, overcame docetaxel-induced autophagy and improved the sensitivity of lung adenocarcinoma (LAD) cells to docetaxel. Docetaxel-induced autophagy was mediated by High-mobility group box 1 (HMGB1) translocation, which participates in the BECN1 PI3K-III core complex formation *via* MEK/ERK1/2 pathway. Indeed, knockdown of *HMGB1* reverted the sensitivity of LAD cells to docetaxel (34).

Sorafenib is a well-known anti-angiogenic agent and remains the standard treatment in advanced unresectable hepatocellular carcinoma (HCC) (35). Over the past decade, several studies have focused on the underlying mechanisms induced by Sorafenib and exploring new combination therapies. In various types of cancers, Sorafenib has been described as inducing autophagy (36). Yuan et al. showed that 3-MA treatment in combination with Sorafenib significantly improved growth inhibition in HepG2, Hep3B, and PLC/PRF/5 treated cells (37). In triple-negative breast cancer (TNBC) cells, dual inhibition of autophagy and the Insulin-like growth factor (IGF) signaling pathway using 3-MA and NVP-AEW541, respectively, enhanced the NVP-AEW541-induced cell growth inhibition and apoptosis (38). In addition, preclinical studies have focused on exploring the benefit of epidermal growth factor receptor (EGFR) inhibitors such as gefitinib in EGFR-positive cancers (39). In TNBC cells, an increasing concentration of gefitinib combined with 3-MA significantly decreased cell viability *in vitro*. Interestingly, in TNBC xenograft mice models, a gefitinib and 3-MA combination resulted in a significant decrease in tumor volume and tumor weight compared to the gefitinib treated group. Further investigations revealed that autophagy inhibition by 3-MA enhanced gefitinib-induced GO/G1 cell cycle arrest, DNA damage, and cell death *via* the mitochondrial apoptosis pathway (40).

Selective VPS34 kinase inhibitors have acquired great interest as potential potent drugs to inhibit early autophagy. The VPS34 kinase inhibitor SAR405 in combination with everolimus, a well-known mTOR inhibitor approved for the treatment of various tumors (41), induced efficient autophagy inhibition and reduced renal tumor cell proliferation *in vitro* (42). In addition, the VPS34 kinase inhibitor SB02024 in combination with sunitinib, a tyrosine kinase inhibitor, significantly decreases cell viability and multicellular spheroid (MCS) growth in both MCF-7 and MDA-MB-231 breast cancer cell lines. Notably, inhibition of MCS growth was not observed with a chloroquine (CQ) and sunitinib combination (43). We have recently shown that pharmacological targeting of VPS34 kinase activity by SB02024 (Sprint Bioscience) or SAR405 (Sanofi) significantly decreased tumor growth and improved mice survival in melanoma B16-F10 and colorectal CT26 tumor mouse models (44). We provided evidence that deep changes in the immune landscape occurred in B16-F10 and CT26 mice models treated with VPS34 inhibitors (SB02024 and SAR405), characterized by increased infiltration of immune effectors such as NK, dendritic cells (DCs), M1 macrophages, and CD8+ T cells in the tumor microenvironment. Because there was no difference in the growth of tumors engrafted in NOD scid gamma (NSG) mice upon treatment with VPS34 inhibitors, these data clearly indicated that the tumor inhibitory effect of VPS34 inhibitors involves the host immune system. Moreover, we demonstrated that pro-inflammatory chemokines such as CCL5 and CXCL10 are responsible for NK and CD8+ T cell recruitment in B16-F10 tumors and CT26 tumors treated with VPS34 inhibitors relative to control. Interestingly, SB02024 or SAR405 improved the therapeutic benefit of anti-PD-1/PD-L1 by significantly reducing tumor growth and tumor weight and improving mice survival in B16-F10 and CT26 tumors (44).

### Inhibition of Beclin-1

It has been reported that genetic inhibition of *BECN1* or *UVRAG* potentiated IR-induced DNA double-strand breaks (DSBs) and cell death in colorectal cancer cells (45). Furthermore, gene silencing of *BECN-1* enhanced the efficiency of fasudil (a RhoA/ROCK inhibitor) to induce apoptosis in esophageal squamous cell carcinoma cells (46). Similarly, *in vitro* suppression of *BECN1* reduces paclitaxel-mediated cell viability, colony formation, and induced apoptotic death in BT-474 and MDA-MB-231 breast cancer cells in dose- and time-dependent manners (47). Similar effects were observed in non-small cancer lung cancer (NSCLC) cells, endometrial carcinoma, nasopharyngeal carcinoma cells, and ovarian and renal cancers (48–53). Interestingly, the therapeutic benefit of paclitaxel was increased in a *Becn1*-targeted BT-474 xenograft mice model based on cleaved caspase-3 positive cells and inhibition of tumor growth (47).

In human chronic myeloid leukemia (CML) cells, co-treatment with spautin-1 and imatinib, a BCR-ABL tyrosine kinase inhibitor, potentiated imatinib-induced CML cell apoptosis in both the K562 cell line and primary cells (54). In line with this latter study, it has also been reported that imatinib in combination with microRNA-30a, identified as potent inhibitor of *BECN1* and *ATG5*, significantly increased the imatinib-mediated cytotoxicity in CML cells (55).

Tamoxifen is one of the most efficient endocrine treatments in estrogen receptor (ER) positive breast cancers, which account for 70% of the breast cancer subtypes. However, the therapeutic benefit of Tamoxifen is negatively impacted by the development of drug resistance (56). Gu et al. discovered that tamoxifen resistance was associated with an increased BECN1 and human epidermal growth factor receptor 2 (HER2) expression in breast cancer cells. *BECN1* silencing enhanced the sensitivity of breast cancer cells to tamoxifen by reducing tumor cell proliferation, migration, and invasion capabilities. These data highlight a novel role of BECN1 in HER2 regulation that contributes to tamoxifen resistance (57).

Using several ovarian cancer cells, Zhang et al. showed that cancer cell resistance to cisplatin relied on autophagy-dependent induction of nuclear accumbens-1 (NAC1). Indeed, targeting *NAC1* or autophagy, *via* 3-MA or *BECN1* silencing, enhanced ovarian cancer cell sensitivity to cisplatin (58). Similar results were reported in adenoid cystic carcinoma of the salivary glands, glioma, and urothelial carcinoma (59–61).

Bevacizumab, a monoclonal anti-vascular endothelial growth factor (VEGF) antibody, is widely used to treat metastatic colorectal cancer, lung cancer and renal cell carcinoma (62–64). Knowing that bevacizumab induces autophagy, it has been reported that genetic inhibition of *BECN1* improves the anticancer effects of this drug in colorectal cancer cells (65). Likewise, improved clinical response to trastuzumab was observed in HER2+ breast cancer displaying loss of *BECN1* gene (38, 66). The role of autophagy, including BECN1, in tumor resistance to targeted therapy is comprehensively reviewed by Mele et al. (67).

We have previously reported that genetic targeting of *Becn1* in melanoma cells prevents the degradation of Natural killer (NK)-derived Granzyme B and enhances melanoma susceptibility to NK-mediated killing (68). Furthermore, we showed that the infiltration of NK cells into *Becn1* defective melanoma is increased, which results in significant inhibition of tumor growth (69). Importantly, the impact of inhibiting autophagy on the infiltration of cytotoxic immune cells into the tumors and the decrease in tumor growth is also reported by other studies (70–73).

## Targeting Autophagosome Maturation Genes ATG4B, ATG5, and ATG7 Potentiates Anticancer Therapies

The third major step of autophagy involves two key complexes that promote the expansion of the phagophore membrane and result in the formation of a double-membraned vesicle named autophagosome. The first complex involves cooperation between ATG4B and ATG7, allowing for the conjugation of LC3I with phosphatidylethanolamine (PE) to form LC3II. LC3II is subsequently incorporated into the newly formed autophagosome (74). The second complex includes ATG7 and the E2-like enzyme ATG10, which are involved in ATG5-ATG12 conjugation (2). In this section, we will describe the therapeutic benefit of inhibiting autophagy genes involved in the maturation of autophagosomes.

### Inhibition of ATG4B

The serine/threonine protein kinase MST4, also known as mammalian STE20-like protein kinase 4 (MST4) (75), was reported to facilitate p-ERK pathway and promote epithelial to mesenchymal transition (EMT) and cancer metastasis in gastric cancer (76). MST4 is associated with prostate cancer, hepatocellular carcinoma, and breast cancer progression (77, 78). It has been reported that MST4 directly phosphorylates ATG4B at S383 position (79). Furthermore, ATG4B inhibition, by NSC185058 (80), improves the anti-tumor effect of radiotherapy in intracranial glioblastoma (GBM) patient derived xenograft (PDX) mice models (79). These data suggest a potential interconnection between MST4, autophagy and malignancy in GBM; however, the value of direct targeting MST4 as a strategy to modulate autophagy remains to be defined.

Recently, benzotropolone derivatives were synthetized and tested for ATG4B inhibition. UAMC-2526 was selected as the best candidate according to its efficiency to reduce basal autophagy and its high stability in the plasma. A combination of UAMC-2526 with oxaliplatin-based chemotherapy reduced colorectal cell proliferation and promoted tumor growth inhibition in HT29 colorectal tumor-bearing mice (81).

FMK-9a is another ATG4B inhibitor, reported to attenuate the pro-LC3 cleavage process and the LC3-PE delipidation. FMK-9a could also induce autophagy independent of its inhibition on ATG4B activity (82). Recently, S130 has been identified to bind and inhibit ATG4B, hence attenuating the delipidation of LC3-II and suppressing the recycling of LC3-I in colorectal cancer cells. Therefore, S130 has been described as a novel small-molecule to improve cancer therapy (83).

Moreover, an FDA-approved drug screening identified tioconazole as a new ATG4 inhibitor. Tioconazole treatment enhanced doxorubicin efficiency by decreasing cell viability in H4, HCT116, and MDA-MB-231 cells. Interestingly, the combination of tioconazole and doxorubicin showed an enhanced antitumor effect in HCT116 xenografted mice relative to each drug alone (84). In this context, it has been reported that the HER2 status was positively correlated with the expression of ATG4B protein. Interestingly, ATG4B silencing was associated with reduced viability of trastuzumab treated HER2+ cells compared to trastuzumab treatment alone (85).

### Inhibition of ATG5 and ATG7

In A549 human lung cancer, overcoming the cytoprotective effect of autophagy induced by cisplatin, *via ATG5* silencing, improves cancer cell apoptosis, as compared to cisplatin treatment alone (86). Dual combination of Epirubicin, a structural analog of doxorubicin, with *ATG5*- or *ATG7*-silencing, significantly reduced cell viability in anthracycline-sensitive and resistant TNBC cells (87). O’Donovan et al. showed that combining both siRNA *BECN1* and *ATG7* decreased cell survival in 5-FU-treated esophageal cancer cells while targeting *BECN1* or *ATG7* alone had no impact (88). Therefore, it appears that targeting different steps of autophagy may be a more appropriate strategy to improve chemotherapy efficacy. In TBNC cells, Wu et al. demonstrated that dual inhibition of *ATG7*, genetically, and IGF-1R pharmacologically, promotes apoptosis and cell growth inhibition (38). In PDAC cells, genetic inhibition of *ATG5* or *ATG7* significantly improved the effect of ERK inhibitors on inhibiting cell proliferation relative to ERK inhibitors alone (30). Dual combination of *ATG5* siRNA and docetaxel, a well-known second-line approved treatment in NSCLC, decreased cell proliferation together with increasing cytotoxicity and apoptosis in LAD cells (34). In renal cell carcinoma cells, *ATG5* silencing or 3-MA treatment in combination with Sorafenib enhanced the sensitivity of RCC cells to Sorafenib (89).

## Impact of Inhibiting Autophagosome-Lysosome Fusion on the Response to Various Anticancer Therapies

The final step of autophagy consists of fusion between autophagosomes and lysosomes for the degradation, and recycling of damaged proteins and organelles. Thus, the outer membrane of autophagosomes merges with the lysosomal membrane, and then the inner membrane is degraded (90). The principal factors involved in autophagosome-lysosome fusion are the homotypic fusion and protein sorting (HOPS) complex, RAB7, and the N-ethylmaleimide-sensitive factor attachment protein receptors (SNAREs) (91). Other proteins, such as the lysosomal-associated membrane protein 2 (LAMP2), are also required for proper fusion (92). This part will focus on the impact of targeting these factors and the fusion step on the therapeutic benefit of radiotherapy, chemotherapy, targeted therapy, and immunotherapy.

### Targeting the Soluble N-Ethylmaleimide-Sensitive Factor Attachment Protein Receptors (SNAREs) to Enhance Response to Conventional Treatments

Syntaxin 17 (STX17), SNAP29, and vesicle-associated membrane protein 8 (VAMP8) are N-ethylmaleimide-sensitive factor attachment protein receptors (SNAREs) involved in the autophagosome-lysosome fusion. During this process, STX17 is recruited to the outer membrane of autophagosomes through HOPS complex and interacts with VAMP8, located on lysosomes. This interaction is enhanced by SNAP29, which forms a SNARE complex with STX17 and VAMP8 (93). Knockdown of *STX17* causes a blockade of the fusion between autophagosomes and lysosomes and results in the accumulation of autophagosomes (93). Therefore, targeting SNARE proteins is considered a strategy for preventing the late step of autophagy. Moreover, SNAP29-STX17-VAMP8 complex formation can be enhanced by the down-regulation of *O*-GlcNAc transferase (OGT). This was correlated with the resistance of ovarian cancer to cisplatin treatment (94), and highlights that targeting SNAP29-STX17-VAMP8 complex by overexpression of OGT could improve the sensitivity to cisplatin treatment. Overexpression of *VAMP8* has also been associated with resistance to temozolomide in human glioma cells, and knockdown of *STX17* in glioma cells overexpressing *VAMP8* led to increased chemosensitivity (95).

In addition, Berbamine, a natural product isolated from traditional Chinese medicine, inhibits autophagosome-lysosome fusion by preventing the interaction between VAMP8 and SNAP29. Berbamine was proposed as a new potential inhibitor of autophagy that could enhance the effects of chemotherapy treatment (96). Moreover, Berbamine has been investigated as a potential anticancer drug in several studies and seems to act on multiple pathways such as MEK/ERK (97) and WNT/β-catenin pathways (98).

### Targeting Lysosomal-Associated Membrane Protein 2 (LAMP2) as a Potential Target to Inhibit Autophagosome-Lysosome Fusion and Improve the Response to Anti-Cancer Therapies

LAMP2 is a glycosylated protein ubiquitously expressed, and mostly located on lysosome membranes. LAMP2 is required for the proper fusion between autophagosomes and lysosomes (92). In neuroendocrine prostate cancer, knockdown of *LAMP2* by siRNA induced an autophagy blockade and decreased both cancer cell proliferation and neuroendocrine markers. These results indicate that LAMP2 plays a dual role in cell survival, by inducing autophagy and in the differentiation of androgen-sensitive human prostate adenocarcinoma cells into neuroendocrine prostate cancer cells (99). In addition, a recent *in silico* approach showed that the expression of *LAMP2* was decreased in prostate cancer tissues as compared to normal prostate tissues (100), indicating that the expression level of *LAMP2* could act as a regulatory element in cancer progression. Another study compared the expression level of *LAMP2* in salivary adenoid cystic carcinoma and pleomorphic adenoma and/or a normal salivary gland (101). The results showed an increased expression of *LAMP2* in salivary adenoid cystic carcinoma, which was associated with cancer progression. Although the expression level of *LAMP2* seems to be different in various cancer types, several data suggest that *LAMP2* is a potential target for cancer therapy in combination with conventional treatments. This statement was supported by data showing that the silencing of *LAMP2* by siRNAs led to a radiosensitization of prostate cancer cell lines (102). In addition, a reduced expression of *LAMP2* has been associated with a decreased resistance to both cisplatin in human ovarian carcinoma cells (103) and azacitidine in acute myeloid leukemia (104). Nevertheless, the sensitization to radiotherapy and chemotherapy by *LAMP2* targeting should be investigated in other types of cancer cells.

### Overexpression of *RAB7* as a Potential Strategy to Improve Sensitivity to Anti-Cancer Treatments

RAB7 is a small GTPase localized to late endosomes and lysosomes and has multiple functions in autophagy. In mammalian, RAB7 is not directly involved in the autophagosome-lysosome fusion but rather in autolysosome maturation under nutrient-rich conditions (105). The significance of RAB7 as a target for autophagy modulation is not well defined so far.

The role of RAB7 in cancer progression has recently been described as a protein involved in promoting the proliferation, invasion, and migration of gastric cancer cells (106). RAB7 has also been associated with chemoresistance to cisplatin. Indeed, *RAB7* was downregulated in cisplatin-resistant cervical cancer cell lines as compared to chemosensitive ones (107). Additionally, *RAB7* overexpression induced chemosensitization of cisplatin-resistant cells, while depletion of *RAB7* by siRNA induced resistance to cisplatin in chemosensitive cells (108). Furthermore, RUBICON (Run domain Beclin-1 interacting and cysteine-rich containing), a negative regulator of autophagy, inhibits autophagosome-lysosome fusion and interacts with RAB7-GTP *via* a RUBICON homology (RH) domain (109).

### Impact of Using Drugs Inhibiting the Last Step of Autophagy Process on Various Cancer Therapies

CQ and its derivate, hydroxychloroquine (HCQ), are the only drugs having shown their ability to block the last step of autophagy and being approved by the Food and Drug Administration (FDA) for clinical use. Indeed, CQ blocks the autophagic flux by altering autophagosome fusion with lysosomes, presumably by interfering with SNAP29 recruitment (110). In addition, CQ cytotoxicity induced autophagy-associated cell death associated with nuclei abnormalities, lipofuscinogenesis, and senescence (111). As reported in clinicaltrials.gov, CQ is currently being investigated as a potent anticancer drug in small cell lung cancer, breast cancer, pancreatic cancer, glioblastoma, melanoma, and other types of cancers.

Because CQ and HCQ are the only autophagy inhibitors available and approved for clinical use, multiple studies evaluated the potential effects of CQ *in vitro*, in combination with anticancer treatments. In fact, CQ was responsible for radio-sensitizing bladder cancer cells and bladder cancer xenografts in mice (112). CQ also had a synergetic effect with radiotherapy on glioma initiating cells by inducing apoptosis and inhibiting autophagy induced by ionizing radiation (113). The same synergistic effects were observed on glioblastoma cell lines (114). CQ administered after radiation is also capable of increasing the death of breast cancer cells and tumor regression *in vivo* (115). The use of CQ as a potent enhancer of radiotherapy is currently being evaluated in clinical trials involving small cell lung cancer (NCT01575782) and glioblastoma (NCT04397679), and in patients with brain metastases from solid tumors (NCT01894633). It should be highlighted that CQ sensitized various breast cancer cell lines to cisplatin and LY294002, reported to induce autophagy in these cells. However, CQ sensitization in these cells occurred independent of autophagy inhibition. Therefore, the autophagy independent sensitizing effects of CQ should be considered in clinical trials where CQ or its derivatives are used in the treatment of cancer (116, 117). In HCT-116 and HT-29 colorectal cancer cell lines, it has been reported that CQ sensitized these cell lines to radiation and 5-FU treatment and resulted in a significant decrease in clonogenic survival of HT-29 cell line without any impact on cell cycle progression or cell death (118). However, in GBM, CQ was found to induce P53-independent cell deaths that do not require caspase-mediated apoptosis. The CQ derivatives, Quinacrine and Mefloquine, are more potent and displayed superior blood-brain barrier penetration compared to CQ (119).

Maycotte et al. evaluated the effects of combining CQ with chemotherapeutic drugs such as the DNA damaging agent cisplatin, the mTOR inhibitor Rapamycin, and the PtdIns3K inhibitor LY294002 in two mouse breast cancer cell lines (117). While the combination of CQ and cisplatin had no significant effect on the viability of both cell lines, CQ combined with PtdIns3K and mTOR inhibition sensitized both cell lines. However, the CQ-mediated sensitization seems to be independent of autophagy, since this sensitization was not observed following *Atg12* and *Becn1* knockdown (117). A similar result was observed in KRAS-driven cancer cell lines where the antiproliferative effects of CQ were similar between *ATG7*-deficient tumor cell lines with undetectable autophagic flux and *ATG7*-efficient tumor cell lines (120). In addition, CQ sensitizes bladder cancer cells to cisplatin treatment by inhibiting cisplatin-induced autophagy (121). Similar results were observed in nasopharyngeal carcinoma cells (122) and hypopharyngeal squamous cell carcinoma xenografted mice (123). This suggests that CQ effects, in combination with chemotherapy, depend on the type of cancer and therefore require further investigation.

Several studies have been conducted on CQ in combination with targeted therapies. Erlotinib and Rapamycin are two tyrosine kinase inhibitors targeting the EGFR and the mammalian target of Rapamycin, respectively. These anticancer drugs are particularly used for NSCLC treatment where EGFR and PI3K/AKT/mTOR pathways are often dysregulated. It has been shown that the combination of Erlotinib and Rapamycin with Monensin, a polyether antibiotic inhibiting autophagosome-lysosome fusion, improved Erlotinib and Rapamycin induced tumor growth inhibition and apoptosis in NSCLC (124). Similar results were observed in prostate cancer cells by Monensin, although the involvement of autophagy inhibition was not clearly suggested in this study (125). Furthermore, the combination of the tyrosine kinase inhibitor, sunitinib, with CQ or *LAMP2* knockdown also showed promising results in a metastatic pancreatic neuroendocrine tumor mice model (126). The combination of the monoclonal antibody trastuzumab with CQ in HER2+ breast cancer (127) also led to promising results. Indeed, CQ sensitized both trastuzumab-resistant breast cancer cells and trastuzumab-resistant xenografts, resulting in increased cell death *in vitro* and decreased tumor growth *in vivo*.

In addition to its effects in combination with radiotherapy, chemotherapy, and targeted therapy, CQ seems to be responsible for various effects on the immune system. CQ resets tumor-associated M2 macrophages to the tumor-inhibiting M1 phenotype in B16 melanoma and H22 hepatocarcinoma mouse tumor models, and ameliorates the immunosuppressive tumor immune microenvironment through a lysosomal calcium-TFEB pathway (91). Another recent article showed that CQ in combination with 5-FU increased the driving of DC maturation by HCT-116 colon cancer cells, and in this way stimulates T cell responses induced by tumor cell lysates (128). Considering the impact of CQ on the immune system, CQ was tested in combination with dual CTLA4 and PD-1 immune checkpoint blockade in orthotopic tumors established from pancreatic ductal adenocarcinoma cells. This study revealed an increased CD8+ T cell infiltration within the tumors and a tumor sensitization to anti-PD-1 and anti-CTLA4 therapy when combined with CQ (129). These data support further investigations on the potential effect of combining CQ with other immunotherapy such as new immune checkpoint inhibitors, T-cell transfer therapy, or monoclonal antibodies, on the immunosuppressive tumor microenvironment.

Besides CQ, Bafilomycin A1 (BafA1) is another drug acting at the last step of autophagy. BafA1 is an antibiotic targeting the vacuolar H+-ATPase enzyme, thus inducing acidification of lysosomal pH, therefore inhibiting lysosomal degradation capacity. BafA1 has also shown promising effects in combination with chemotherapy, since BafA1 increased cisplatin cytotoxicity in tongue squamous cell carcinoma cells and bladder cancer cells (121, 130). BafA1 also increased chemosensitivity to 5-FU in gastric cancer cells (131). Other drugs have been described to target autophagosome-lysosome fusion, such as LS-1-10, Cytochalasin E and Simvastatin. LS-1-10 is a novel acridine derivative sharing structure with CQ and HCQ. LS-1-10 has a dual function, it is able to both induce DNA damage and block autophagosome-lysosome fusion. In addition, LS-1-10 is significantly more potent in reducing cell viability than CQ (>50%) in colon cancer cells (132).

Cytochalasin E is a fungal toxin found in *Aspergillus clavatus*, which binds to actin filaments. Cytochalasin E can inhibit autophagosome-lysosome fusion in the same way as CQ. In addition, cytochalasin enhances the effect of bortezomib in human lung cancer cells (133). Simvastatin is a powerful inhibitor of hydroxymethylglutaryl CoA reductase, an enzyme involved in cholesterol biosynthesis. Interestingly, Simvastatin can induce cell death in astrocytoma, neuroblastoma, glioblastoma, breast cancer, and lung adenocarcinoma (134). Moreover, this drug appears to inhibit the fusion between autophagosomes and lysosomes and increase the effects of Temozolomide in glioblastoma cell lines (135). Finally, the combination of Simvastatin with Vorinostat, a histone deacetylase inhibitor, inhibits autophagy by preventing RAB7 prenylation and decreases tumor growth in mice bearing triple-negative breast tumors (136).

## Conclusion

Despite the dual role of autophagy in suppressing tumor initiation and in promoting the survival of established tumors (16), the studies reported in this review highlight the pivotal role of autophagy as a cytoprotective and therapy resistance mechanism in cancer. Therefore, strategies used to modulate autophagy for enhancing the therapeutic benefit of current anticancer therapies are an area of intense investigation. Based on data reported in this review, we strongly believe that inhibiting autophagy represents a new paradigm for overcoming therapy resistance and enhancing drug sensitivity in multiple tumor cell types. Because autophagy inhibition is currently used in many clinical trials along with different therapeutic strategies, it is reasonable to consider that several cancer treatments themselves induce autophagy in tumor cells.

Among the various autophagy inhibitors, CQ or its derivative HCQ are the major drugs used in clinical trials, with mitigated success. While CQ provides promising results in combination with conventional anticancer therapies, in some studies CQ sensitization appears to be independent of autophagy inhibition. Therefore, more selective and potent autophagy inhibitors must be designed to definitively endorse the therapeutic benefit of targeting autophagy in cancer patients. While CQ and HCQ inhibits the last step of autophagy (110), other druggable autophagy proteins have recently been proposed, which include the early autophagy protein BECN1 (45) and its interacting protein VPS34 (or PI3K class III) (41, 42, 44). Thus, more potent and selective autophagy specific inhibitors are currently in pre-clinical development; these include drugs targeting earlier steps in the autophagy process, such as ULK1, VPS34, and ATG4B (27, 28) (79). Other factors interacting with the autophagy process could also be considered as potential targets to inhibit autophagy and overcome therapy resistance, such as AMPK and HIF-1α, which are reported as key inducers of autophagy through negative regulation of the mTOR pathways and inducing hypoxic conditions, respectively (6, 7, 137). Other drugs are able to target the late step of autophagy by interacting with lysosomes. ROC-325 has been described as a potent autophagy inhibitor exhibiting superior *in vitro* and *in vivo* anticancer effects compared to CQ. In Renal cell carcinoma RCC, ROC-325 induced an accumulation of autophagosomes *in vitro* and inhibited RCC growth and survival in an ATG5/7-dependent manner *in vivo* by disrupting autophagic degradation (138). In acute myeloid leukemia, ROC-325 improved the anti-leukemic activity of azacitidine through inhibiting autophagy (139). BRD1240 is a small-molecule suppressing the V-ATPase function and therefore inhibiting the lysosomal acidification property. It has been reported that, similar to BafA1, BRD1240 inhibited autolysosome formation and subsequently triggering a significant accumulation of autophagosomes (140). Similar to ROC-325 and LS-1-10, Betulinic acid (BA) disrupt the degradative lysosomal function (74, 141–143), leading to the accumulation of mitochondria inside dysfunctional autolysosomes. Such a lysosome-mitochondrial stress axis is responsible for the induction of lipofuscinogenesis and ageing (143).

It should be highlighted that even if a selective and potent autophagy inhibitor is identified, the challenging task is to demonstrate that the therapeutic benefit that could be observed is indeed related to inhibition of the autophagy process, as almost all autophagy-related genes have non-autophagic functions. Another challenge is to maintain a balance between the benefits gained by autophagy inhibition and the deleterious effects of this inhibition in cancer patients. Indeed, the process of autophagy seems to both activates and inhibits cellular senescence (144), and chronic inhibition of autophagy appears to increase permanently the risk of cancer (145). Finally, considering the controversial role of autophagy regarding its cytoprotective or cytotoxic function, it is more likely obvious that the clinical outcome of combination treatment between an inhibitor of autophagy with chemotherapy, radiotherapy, targeted therapy, or immunotherapy would lead to contradictory or equivocal results (146). Therefore, the last challenge to overcome would be to determine whether patients would benefit from autophagy inhibition prior to conventional therapies, with for example the use of novel biomarkers of cytoprotective autophagy.

## Author Contributions

MX, AB, MH, CD, GV, GB, MN, and BJ have contributed in writing and editing the manuscript. BJ has generated figures. All authors contributed to the article and approved the submitted version.

## Funding

This work was supported by Luxembourg Institute of Health and grants from the Luxembourg National Research Fund (PRIDE15/10675146/CANBIO and C18/BM/12670304/COMBATIC); FNRS Televie (grants 7.4606.18 and 7.4579.20); Fondation Cancer, Luxembourg (FC/2018/06); Kriibskrank Kanner Foundation, Luxembourg (2019); RCMS foundation, and Action LIONS Vaincre le Cancer Luxembourg. The authors also declare that this study received funding from Janssen Cilag Pharma and Roche Pharma. These funders were not involved in the study design, collection, analysis, interpretation of data, the writing of this article or the decision to submit it for publication.

## Conflict of Interest

The authors declare that the research was conducted in the absence of any commercial or financial relationships that could be construed as a potential conflict of interest.
